# Clinical characteristics, molecular reclassification trajectories and DNA methylation patterns of long- and short-term survivors of WHO grade II and III glioma

**DOI:** 10.1007/s00415-025-12923-6

**Published:** 2025-02-15

**Authors:** Maximilian J. Mair, Annette Leibetseder, Gerwin Heller, Erwin Tomasich, Lisa Müller, Ilka Busse, Adelheid Wöhrer, Barbara Kiesel, Georg Widhalm, Franziska Eckert, Serge Weis, Josef Pichler, Matthias Preusser, Anna S. Berghoff

**Affiliations:** 1 Division of Oncology, Department of Medicine I, Medical University of Vienna, Waehringer Guertel 18-20, Vienna, Austria; 2 Department of Neurology 1, Neuromed Campus, Kepler University Hospital, Johannes Kepler University Linz, and Clinical Research Institute for Neuroscience, Linz, Austria; 3 Division of Neuropathology and Neurochemistry, Department of Neurology, Medical University of Vienna, Vienna, Austria; 4 Department of Neurosurgery, Medical University of Vienna, Vienna, Austria; 5 Department of Radiation Oncology, Medical University of Vienna, Vienna, Austria; 6 Division of Neuropathology, Department of Pathology and Molecular Pathology, Neuromed Campus, Kepler University Hospital, and Clinical Research Institute for Neuroscience, Johannes Kepler University Linz, Linz, Austria; 7 Department of Internal Medicine and Neurooncology, Neuromed Campus, Kepler University Hospital, Johannes Kepler University Linz, Linz, Austria

**Keywords:** Glioma, Astrocytoma, Oligodendroglioma, Survival, DNA methylation

## Abstract

**Purpose:**

The prognosis of diffuse gliomas previously classified as “lower-grade” is heterogeneous and complicates clinical decisions. We aimed to investigate the molecular profile of clinical outliers to gain insight into biological drivers of long and short-term survivors.

**Methods:**

Here, patients aged ≥ 18 years and diagnosed with diffuse glioma, WHO grade II/2 or III/3 were included. Short-term survivors (STS) were defined as overall survival (OS) < 1 years, and long-term survivors (LTS) as OS > 10 years. DNA methylation profiling was performed using the Illumina EPIC 850k platform.

**Results:**

In total, 385 patients (294 LTS, 91 STS) were included. Median overall survival was 234 months (95%CI: 207–248) in LTS and 7.3 months (95%CI: 6.4–8.1) in STS. Compared to STS, LTS were younger, had higher Karnofsky Performance Status, more extensive resections, and lower symptomatic burden (p < 0.001, respectively). Molecular reclassification showed *IDH-*mutant gliomas in 240/246 (95.5%) LTS and 10/79 (12.7%) STS. Initial diagnosis (tumor type and/or grading) changed in 69/325 (21.2%) patients based on reclassification according to WHO 2016 and in 45/258 (17.4%) as per WHO 2021. DNA methylation analysis indicated two clusters, one with mainly STS (39/41, 95.1%) and heterogeneous *IDH-*wildtype tumors (cluster A) and one with mainly LTS (82/106, 77.4%) and *IDH*-mutant tumors (cluster B). Functional enrichment analysis of rare subtypes indicated altered Hippo/Notch and synaptic/neurotransmitter signaling pathway members.

**Conclusion:**

LTS and STS show distinct clinical and molecular features, underscoring the importance of extended molecular workup for diagnosis. Further characterization of rare subtypes is needed to optimize treatment strategies and clinical trial planning.

**Supplementary Information:**

The online version contains supplementary material available at 10.1007/s00415-025-12923-6.

## Introduction

Diffuse gliomas are a highly heterogeneous group of malignant brain tumors. In line, survival ranges from a few weeks to over a decade, challenging the optimal sequencing of neurotoxic therapies and the planning of inclusion criteria in clinical trials. Recently, molecular factors were included in the revised WHO Classifications of Central Nervous System Tumours in 2016 and 2021, moving from a histopathological classification towards an integrated framework considering both morphological appearance and molecular alterations [1, 2]. In addition, whole-genome DNA methylation profiling is increasingly applied in brain tumor classification, as it allows for the definition of more homogenous tumor entities [3] and also led to the discovery of novel subgroups with distinct biological and clinical characteristics [4–6].

Thereby, molecular characterization including DNA methylation profiling also allowed for improved prognostic stratification [7–12]. However, the rapid evolution of tumor classification frameworks underscores the necessity of continuous validation of their clinical relevance and prognostic impact in large real-life cohorts, guiding further development of brain tumor classification criteria. Indeed, the increasing granularity of brain tumor classification comes with a scarcity of clinical annotation, which is a pivotal basis for treatment decisions, clinical trial planning and the counseling of patients as well as their caregivers. For instance, previous studies establishing the treatment standards for glioblastoma (CNS WHO grade 4) as well as isocitrate dehydrogenase (*IDH*)-mutant glioma (astrocytoma and oligodendroglioma) did not address in full detail the now established insights on molecular markers in the diagnostic workup [13–16]. In consequence, the current treatment recommendations are based on heterogeneous trial cohorts, and several molecular markers were only analyzed in a post-hoc manner [17, 18].

To gather real-life insights on the clinical and molecular drivers of prognostic outliers, we analyzed clinical characteristics, molecular reclassification trajectories and DNA methylation profiles in a large real-life cohort of long- (LTS) and short-term survivors (STS) of tumors previously classified as WHO grade II and III glioma who were managed at two academic neuro-oncology centers in Austria.

## Materials and methods

### Patient cohort

In this retrospective study, adult (≥ 18 years) patients diagnosed in clinical routine with a WHO grade II or III diffuse glioma between 2000 and 2019 and treated at the Medical University of Vienna (Vienna, Austria) or the Kepler University Hospital Linz/Neuromed Campus (Linz, Austria) have been included. Inclusion of patients with recurrent tumor but first histological diagnosis before 2000 was allowed. LTS and STS were defined as patients with an OS (from first radiological suspicion of intracranial tumor) of > 10 or ≤ 1 year(s), respectively. As control group in DNA methylation analyses, also medium-term survivors (MTS) with an OS between 1 and 10 years were included. Histological tumor classification at diagnosis was performed by a board-certified neuropathologist, and molecular reclassification was done based on the 2016/2021 WHO Classification of Tumours of the Central Nervous System and recommendations of the Consortium to Inform Molecular and Practical Approaches to CNS Tumor Taxonomy—Not Official WHO (cIMPACT-NOW). In specific, *IDH* mutations were determined using immunohistochemistry (IHC) for the canonical *IDH1* R132H mutation (anti-IDH1 R132H antibody, clone H09, Dianova GmbH, Hamburg, Germany) or sequencing, which was obligatory in the absence of positive IDH1-R132H IHC and a patient age below 55 years. Codeletions of chromosome arms 1p and 19q were determined using multiplex ligation-dependent probe amplification (MLPA), fluorescent/chromogenic in situ hybridization (FISH/CISH) or DNA methylation analysis (see below). In cases where the detection of 1p/19q codeletion was not feasible, the diagnosis of astrocytoma, *IDH-*mutant was attributed to tumors in the presence of (1) *IDH *mutations, (2) clear astrocytic histology and (3) loss of ATP-dependent helicase ATRX and/or strong nuclear positivity for p53 in accordance with cIMPACT-NOW update 2 [19].

Data were retrieved by review of electronic medical records, and extent of resection (gross-total resection [GTR] vs. subtotal resection [STR] vs. biopsy) was determined based on postoperative magnetic resonance imaging (where available) or surgical notes. All data were entered into a FileMaker-based database (FileMaker Pro Advanced/Server 19, FileMaker Inc., Santa Clara, CA, USA), and all statistical analysis was performed in pseudonymized form. This study was approved by the Ethics Committees of the Medical University of Vienna (protocol no. 1166/2019, 2290/2020) and the Kepler University Hospital Linz (protocol no. 1274/2019). The study was performed in compliance with the Declaration of Helsinki and its amendments as well as according to institutional and national guidelines.

### DNA methylation analysis

Whole-genome DNA methylation analysis was performed on formalin-fixed, paraffin-embedded tissue (preferably from first surgery) retrieved from the Neuro-Biobank of the Medical University of Vienna and the tissue archive of the Division of Neuropathology at the Kepler University Hospital Linz. DNA retrieval, methylation profiling and bioinformatic analyses have been performed as described previously [20]. Heatmaps were created based on beta values representing the proportion of methylated DNA at a given CpG site relative to the total DNA (both methylated and unmethylated) at that location. DNA methylation-based reclassification was performed using the Heidelberg Methylation Classifier (version 12b6) [3]. Copy number variation (CNV) estimation based on EPIC methylation values were determined using the R package *conumee*. The output of these analyses (*.bins.igv files) was used for CNV load calculation as described recently [21]. In silico functional enrichment analyses have been performed using the top 2000 differentially methylated CpG sites mapped to genes using the ShinyGO tool [22].

### Statistical analysis

Statistical analysis was performed using R 4.2.1 (The R Foundation for Statistical Computing, Vienna, Austria) with the packages *readr, survival, survminer, ggpubr, doBy, readxl, ggplot2, ggalluvial,* and *ggrepel* as well as GraphPad Prism 10 (GraphPad, La Jolla, CA, USA). Chi-square and Fisher’s exact test were used to assess independence of categorical variables. Mann–Whitney-U test was performed to compare distributions of numerical variables between groups. OS was defined as the time between first radiological suspicion of intracranial tumor and death or last-follow-up as appropriate. Statistical significance was defined as p ≤ 0.05. Due to the hypothesis-generating scope of the study, no correction for multiple testing was performed [23].

## Results

### Patient cohort

**Fig. 1:**
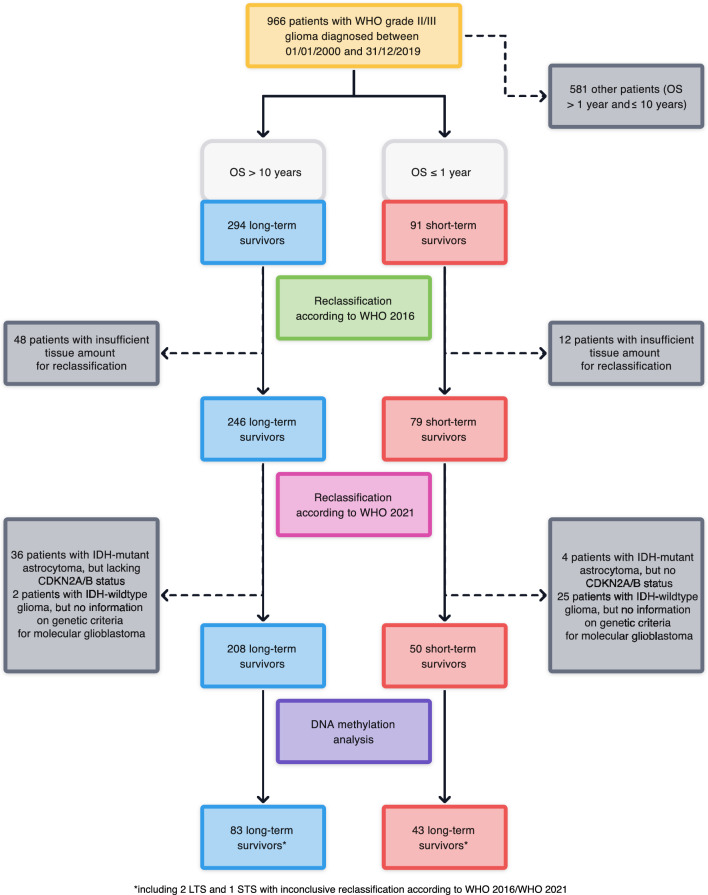
CONSORT-like diagram showing patient disposition and numbers of reclassified tumors. Abbreviations: CDKN2A/B = cyclin-dependent kinase inhibitors 2A/B; IDH = isocitrate dehydrogenase; OS = Overall survival; WHO = World Health Organization

**Fig. 2:**
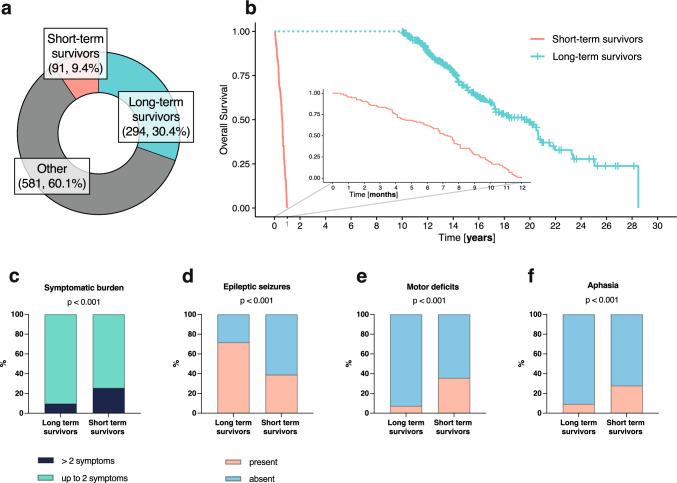
Patient characteristics and symptomatic burden. **a** Frequency of long- and short-term survival in overall cohort; **b** overall survival in included patients (overlaid plot showing survival of short-term survivors within 12 months); **c** symptomatic burden (≤ 2 and > 2 symptoms at presentation), presence of **d** epileptic seizures, **e** motor deficits and **f** aphasia in long- and short-term survivors at presentation. P-values as determined by log-rank, Chi-square or Fisher’s exact test as appropriate

Overall, data of 966 patients diagnosed between 01/01/2000 and 31/12/2019 with histological diagnosis of WHO grade II or III glioma were available, of whom 385 (39.9%) met the inclusion criteria (Fig. 1). In total, 294/966 (30.4%) were LTS and 91/966 (9.4%) were STS (Fig. 2a). Median survival in the LTS cohort was 234 months (95% confidence interval [95%CI]: 207–248), and 7.3 months (95%CI 6.4–8.1) in the STS cohort (Fig. 2b). The clinical characteristics between the STS and LTS cohort differed as LTS were younger (median age: 37 years, range: 18–78) than STS (median age: 65 years, range: 18–81; p < 0.001) and had a higher performance status (median Karnofsky Performance Status [KPS] in LTS: 90% [range: 70%−100%] vs. STS: 80% [range: 40%−100%]; p < 0.001, Mann–Whitney-U). Most tumors in LTS affected the frontal (169/294, 57.5%) and temporal lobes (73/294, 24.8%), whereas tumor sites were more diverse in STS (p < 0.001, Fisher’s exact test). Tumor resections were more extensive in LTS (GTR: 116/298 [39.5%] vs. STR/biopsy: 169/294 [54.1%]) than in STS (GTR: 9/91 [9.9%] vs. STR/biopsy: 80/91 [87.9%]; p < 0.001). A watch-and-wait approach after surgery was followed more frequently in LTS (177/294, 60.2%) than in STS (33/91, 36.3%; p < 0.001). Detailed baseline and treatment characteristics are given in Table 1.

**Table 1:** Patient characteristics. P-values as determined by Chi-square, Fisher's exact or Mann-Whitney-U tests as appropriate

	LTS (n = 294)	STS (n = 91)	p-value
Sex			
Female	140 (47.6%)	39 (42.9%)	0.499
Male	154 (52.4%)	52 (57.1%)	
Median age (years, range) at diagnosis	37 (18–78)	65 (18–81)	** < *0.001***
Median Karnofsky Performance Status (range)	90% (70%−100%)	80% (40%−100%)	** < *0.001***
Original histological diagnosis at first surgery (including pre-WHO 2016 diagnoses)			
Astrocytic	130 (44.2%)	73 (80.2%)	** < *0.001***
Oligodendroglial	86 (29.3%)	8 (8.8%)	
Mixed	78 (26.5%)	8 (8.8%)	
Not otherwise specified (NOS)	0 (0.0%)	2 (2.2%)	
Original WHO grade at first surgery			
WHO grade II	201 (68.4%)	22 (24.2%)	** < *0.001***
WHO grade III	93 (31.6%)	69 (75.8%)	
MGMT promoter methylation status	*n = 165*	*n = 59*	
Methylated	139 (84.2%)	22 (37.3%)	** < *0.001***
Unmethylated	26 (15.8%)	37 (62.7%)	
Tumor site			
Frontal	169 (57.5%)	20 (22.0%)	** < *0.001***
Temporal	73 (24.8%)	24 (26.4%)	
Parietal	16 (5.4%)	15 (16.5%)	
Occipital	9 (3.1%)	4 (4.4%)	
Insular	18 (6.1%)	3 (3.3%)	
Other (supratentorial)	8 (2.7%)	16 (17.6%)	
Other (infratentorial)	1 (0.3%)	9 (9.9%)	
Extent of resection at first surgery			
Gross total resection (GTR)	116 (39.5%)	9 (9.9%)	** < *0.001***
Subtotal resection (STR)	117 (39.8%)	25 (27.5%)	
Biopsy	52 (17.7%)	55 (60.4%)	
*unknown*	*9 (3.1%)*	*2 (2.2%)*	
Postoperative treatment			
Radio-chemotherapy	59 (20.1%)	35 (38.5%)	** < *0.001***
Radiotherapy only	45 (15.3%)	12 (13.2%)	
Chemotherapy only	11 (3.7%)	9 (9.9%)	
Watch-and-wait	177 (60.2%)	33 (36.3%)	
*Unknown/lost to follow-up*	2 (0.7%)	2 (2.2%)	
Chemotherapy regimen	*n* = *70*	*n* = *44*	
RT/TMZ + TMZ	38 (54.3%)	17 (38.6%)	***0.030***
RT/TMZ	4 (5.7%)	12 (27.3%)	
Adjuvant TMZ (without radiotherapy)	9 (12.9%)	6 (13.6%)	
Dose-dense TMZ	6 (8.6%)	5 (11.4%)	
CCNU	6 (8.6%)	2 (4.5%)	
PCV	3 (4.3%)	0 (0.0%)	
Experimental	3 (4.3%)	1 (2.3%)	
RT/TMZ + TMZ (hypofractionated RT)	0 (0.0%)	1 (2.3%)	
*Unknown*	1 (1.4%)	0 (0.0%)	

CCNU = lomustine; LTS = long-term survivors; PCV = procarbazine, CCNU, vincristine; RT = radiotherapy; STS = short-term survivors; TMZ = temozolomide; WHO = World Health Organization

Data on symptoms were available in 292/294 (99.3%) LTS and 90/91 (98.9%) STS. Overall, symptomatic burden was higher in STS (23/90 [25.6%] patients with > 2 symptoms) compared to LTS (28/292 [9.6%]; p < 0.001, Chi-square test, Fig. 2c). Epileptic seizures were more prevalent in LTS (209/292, 71.6%) compared to STS (35/90, 38.9%; p < 0.001, Chi-square test; Fig. 2d). In contrast, STS more frequently experienced motor deficits (32/90 [35.6%] vs. 21/292 [7.2%], p < 0.001) and aphasia (25/90 [27.8%] vs. 27/292 [9.2%], p < 0.001; Fig. 2e/f). Further data on symptoms at presentation are given in Supplementary Fig. 1.

### Molecular reclassification according to WHO 2016 and WHO 2021

Based on the availability of tumor tissue, molecular reclassification according to WHO 2016 could be performed in 325/385 (84.4%) cases. Of these, 246/325 (75.7%) were LTS and 79/325 (24.3%) STS (Fig. 1, Supplementary Fig. 2).

An overview on integrated diagnoses in LTS and STS according to different classification frameworks is given in Table 2. Among LTS, 240/246 (97.6%) had *IDH*-mutant tumors of whom molecular reclassification showed a change of tumor type (such as astrocytoma to oligodendroglioma or vice versa) in 21 (8.8%) patients, while there were no changes in tumor grading. Reclassification of 65 oligoastrocytomas resulted in 32 (49.2%) oligodendrogliomas and 33 (50.8%) astrocytomas according to WHO 2016. Tumor type remained unchanged in 154/240 (64.2%) cases of *IDH-*mutant LTS. In addition, 6/246 (2.4%) LTS had *IDH-*wildtype glial tumors.

**Table 2:** Reclassification according to prognosis

	Histological diagnosis	Total	LTS	STS
Original histological diagnosis (n = 385)	Astrocytic, WHO grade II	104	88 (84.6%)	16 (15.4%)
	Astrocytic, WHO grade III	99	42 (42.4%)	57 (57.6%)
	Mixed (oligoastrocytic), WHO grade II	64	61 (95.3%)	3 (4.7%)
	Mixed (oligoastrocytic), WHO grade III	22	17 (77.3%)	5 (22.7%)
	Oligodendroglioma, WHO grade II	60	58 (96.7%)	2 (3.3%)
	Oligodendroglioma, WHO grade III	34	28 (82.4%)	6 (17.6%)
	Glioma, NOS	2	–	2 (100.0%)
WHO 2016 (n = 325)	Diffuse astrocytoma, *IDH*-mutant, WHO grade II	97	93 (95.9%)	4 (4.1%)
	Anaplastic astrocytoma, *IDH*-mutant, WHO grade III	36	34 (94.4%)	2 (5.6%)
	Oligodendroglioma, *IDH*-mutant, 1p/19q-codeleted, WHO grade II	80	79 (98.8%)	1 (1.3%)
	Anaplastic oligodendroglioma, *IDH*-mutant, 1p/19q-codeleted, WHO grade III	37	34 (91.9%)	3 (8.1%)
	Diffuse astrocytoma, *IDH*-wildtype, WHO grade II	18	4 (22.2%)	14 (77.8%)
	Anaplastic astrocytoma, *IDH*-wildtype, WHO grade III	52	1 (1.9%)	51 (98.1%)
	Diffuse midline glioma, *H3K27*-mutant, WHO grade IV	2	–	2 (100.0%)
	Anaplastic pilocytic astrocytoma, WHO grade III	2	–	2 (100.0%)
	Dysembryoplastic neuroepithelial tumor, WHO grade I	1	1 (100.0%)	-
WHO 2021 (n = 258)	Astrocytoma, *IDH*-mutant, CNS WHO grade 2	66	64 (97.0%)	2 (3.0%)
	Astrocytoma, *IDH*-mutant, CNS WHO grade 3	24	24 (100.0%)	–
	Astrocytoma, *IDH*-mutant, CNS WHO grade 4	3	3 (100.0%)	–
	Oligodendroglioma, *IDH*-mutant, 1p/19q-codeleted, CNS WHO grade 2	80	79 (98.8%)	1 (1.2%)
	Oligodendroglioma, *IDH*-mutant, 1p/19q-codeleted, CNS WHO grade 3	37	34 (91.9%)	3 (8.1%)
	Glioblastoma, *IDH*-wildtype, CNS WHO grade 4	42	3 (7.1%)	39 (92.9%)
	Diffuse midline glioma, *H3K27*-altered, CNS WHO grade 4	2	–	2 (100.0%)
	High-grade astrocytoma with piloid features, CNS WHO grade 3	3	–	3 (100.0%)
	Dysembryoplastic neuroepithelial tumor, CNS WHO grade 1	1	1 (100.0%)	–

CNS = Central Nervous System; IDH = isocitrate dehydrogenase; LTS = long-term survivors; NOS = not otherwise specified; STS = short-term survivors; WHO = World Health Organization

In STS, 69/79 (87.3%) tumors were *IDH-*wildtype, including 65 (94.2%) astrocytomas, *IDH-*wildtype. Newly assigned tumor types according to reclassification were seen in 4 cases, including 2 (2.9%) diffuse midline gliomas, *H3K27-*mutant (with resulting change in grading to WHO grade IV) and 2 (2.9%) anaplastic astrocytomas with piloid features. Interestingly, 10/79 (12.7%) STS had *IDH-*mutant gliomas, of whom 6 were reclassified as astrocytoma, *IDH-*mutant and 4 as oligodendroglioma, *IDH-*mutant, 1p/19q-codeleted according to WHO 2016.

Further reclassification according to WHO 2021 was performed in 258 patients. In the remaining cases, information on *CDKN2A/B* status was missing in 40 patients with *IDH-*mutant astrocytoma, and information on molecular features of glioblastoma (*TERT* mutation, *EGFR* amplification, 7p + /10q-) was missing in 27 patients with *IDH-*wildtype glioma. Overall, there was a reclassified tumor type in 42 samples (39 STS, 3 LTS), which were previously classified as astrocytoma, *IDH-*wildtype and fulfilled molecular criteria of glioblastoma. Consequently, assigned tumor grade increased to CNS WHO 4 in 10 samples previously graded as WHO grade II and 32 samples with WHO grade III. In addition, 3 samples with astrocytoma, *IDH-*mutant (WHO grade II: 1; WHO grade III: 2) were graded as CNS WHO grade 4 as they harbored homozygous deletions of *CDKN2A/B* (all LTS).

Of note, 2 patients with astrocytoma (CNS WHO grade 2) and 4 patients with oligodendroglioma (1 with CNS WHO grade 2; 3 with CNS WHO grade 3) were STS. In contrast, 3 patients with glioblastoma according to WHO 2021 were LTS.

### Unsupervised clustering of DNA methylation profiles

**Fig. 3:**
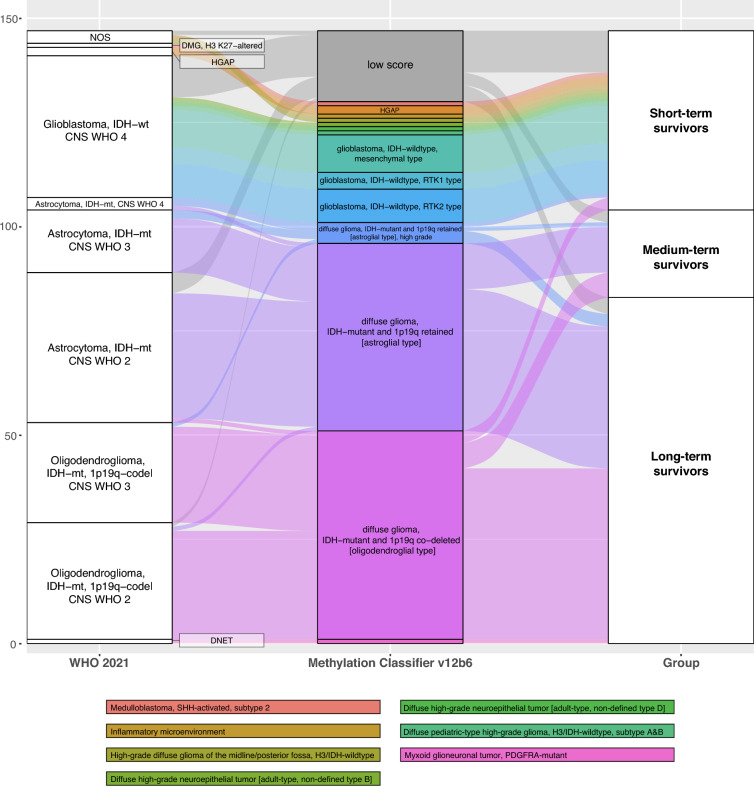
Alluvial plot showing DNA methylation-based reclassification in 147 long-, medium-, and short-term survivors. Legend below showing methylation classification of small subgroups (from top to bottom). Abbreviations: CNS = Central Nervous System; codel = codeleted; DMG = Diffuse midline glioma, *H3K27*-altered; DNET = dysembryoplastic neuroepithelial tumor; HGAP = high-grade astrocytoma with piloid features; IDH = isocitrate dehydrogenase; mt = mutant; NOS = not otherwise specified; PDGFRA = platelet-derived growth factor receptor A; RTK1/2 = subclass receptor tyrosine kinase I/II; SHH = sonic hedgehog; WHO = World Health Organization; wt = wildtype

To further investigate biological alterations in LTS and STS, especially considering clinical “outliers” with unexpected prognosis based on their integrated diagnosis, we performed DNA methylation analysis in 126/389 (32.4%) patients of the cohort (LTS: 83/126 [65.9%]; STS: 43/126 [34.1%]) and included further 21 medium-term survivors (MTS). Further baseline characteristics of this cohort are given in Supplementary Table 1, and reclassification according to the Heidelberg Molecular Neuropathology Classifier (MC) version 12b6 is shown in Fig. 3.

**Fig. 4:**
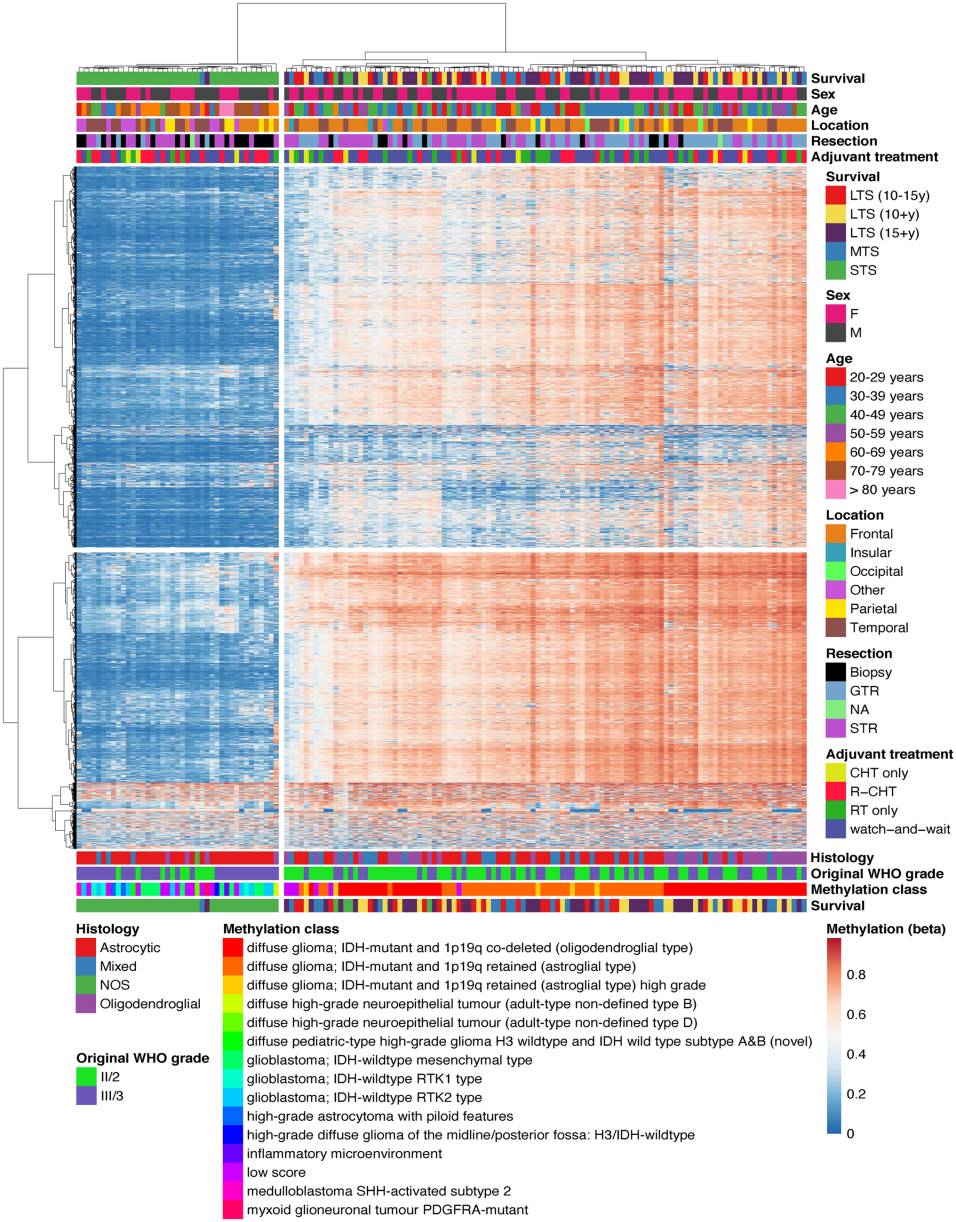
Heatmap and unsupervised clustering analysis based on DNA methylation analysis including clinical factors. Heatmap based on beta values reflecting the proportion of methylated DNA at a given CpG site relative to the total DNA (both methylated and unmethylated) at that location. Abbreviations: CHT = chemotherapy; F = female; GTR = gross total resection; LTS (10-15y) = long term survivors with an overall survival of 10–15 years (deceased); LTS (10 + y) = long term survivors with an overall survival of 10–15 years (censored); LTS (15 + y) = long term survivors with an overall survival of more than 15 years; M = male; NA = not available; R-CHT = radio-chemotherapy; RT = radiotherapy; STR = subtotal resection; STS = short term survivor; WHO = World Health Organization

By unsupervised clustering (Fig. 4), two clusters were obvious, with cluster A consisting of predominantly STS (39/41. [95.1%]), and cluster B comprising mainly LTS (82/106, [77.4%]) and MTS (20/106 [18.9%]) as well as *IDH-*mutant tumors (100/106, [94.3%]).

Interestingly, one LTS and one MTS were found in cluster A, and 4 STS were found in cluster B. The methylation profile of one LTS in the predominantly STS cluster A was compatible with myxoid glioneuronal tumour, *PDGFRA*-mutant, while the classifier score of the MTS case in the predominantly STS cluster A was below the threshold (“low score”).

STS in the predominantly LTS cluster B comprised three cases with oligodendroglioma (CNS WHO grade 3), of whom one displayed a CNV profile suggesting complete loss of chromosome 9 (including the locus for *CDKN2A/B)*, while another tumor harbored losses in chromosomes 14q, 15q, and gain in 11q. The remaining STS case in the predominantly LTS cluster B was previously classified as astrocytoma (CNS WHO grade 2) as MLPA did not show 1p/19q codeletion as predicted by DNA methylation profiling.

According to classifier version 12b6, a different diagnosis compared the WHO 2021 framework was assigned in 9/147 (6.1%) cases, mainly in *IDH-*wildtype tumors showing diverse methylation classifier diagnoses. Clinical characteristics of patients according to these methylation classes are given in Supplementary Table 2.

A correlation between total CNV load and prognosis has been reported in astrocytoma previously [24]. However, in our cohort, no association between OS and total CNV load was observed (Supplementary Fig. 3).

### Functional enrichment analyses of rare subtypes

To investigate which molecular pathways may be altered in rare, newly assigned DNA methylation-based diagnoses compared to WHO classifications, we compared their DNA methylation profiles with the largest subgroup in each cohort (*IDH-*mutant glioma in LTS, glioblastoma in STS).

**Fig. 5:**
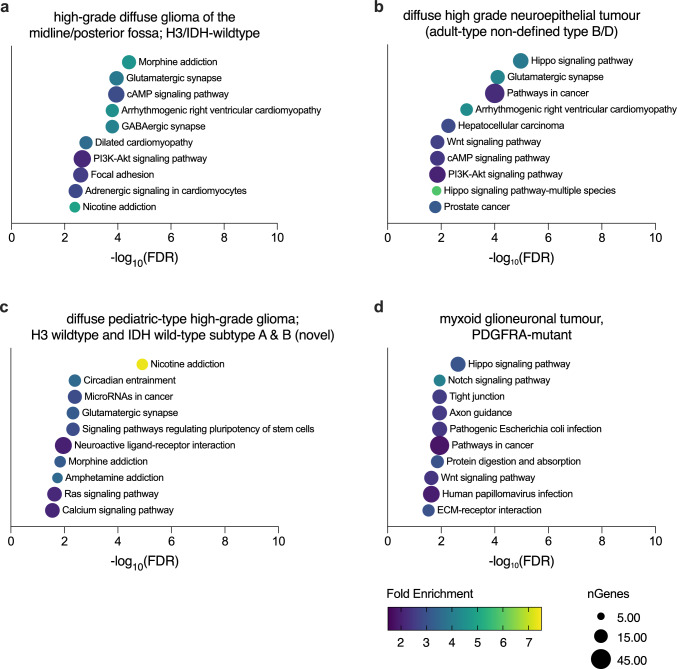
Functional enrichment analysis in **a** high-grade diffuse glioma of the midline/posterior fossa; H3/IDH-wildtype, **b** diffuse high-grade neuroepithelial tumor (adult-type non-defined type B/D), **c** diffuse pediatric-type high-grade glioma, H3 wildtype and IDH wildtype subtype A & B (novel), and **d** myxoid glioneuronal tumor. Top 10 enriched categories according to false discovery rate (FDR) compared to (**a/b/c**) glioblastoma (*IDH-*wildtype) as reference group for short-term survivors and (**d**) *IDH-*mutant gliomas as reference group for long-term survivors

In STS, these rare subtypes included high-grade diffuse glioma of the midline/posterior fossa: H3/IDH-wildtype; diffuse high-grade neuroepithelial tumor (adult-type, non-defined types B and D); and diffuse pediatric-type high-grade glioma, H3 wildtype and *IDH*-wildtype, subtype A&B (novel). Cluster analysis showed distinct methylation profiles compared to glioblastoma (Supplementary Fig. 4a-c). Pathway enrichment analyses based on the Kyoto Encyclopedia of Genes and Genomes (KEGG) showed several altered molecular pathways involving synaptic signaling as well as neurotransmitter and Hippo signaling pathways (Fig. 5a–c).

In LTS, myxoid glioneuronal tumor, *PDGFRA-*mutant was seen as re-assigned LTS entity which clustered in the predominantly STS cluster A. In line, clustering showed a distinct methylation profile from *IDH-*mutant glioma as comparison group for LTS (Supplementary Fig. 3d). Also here, functional enrichment analysis revealed Hippo signaling among other altered molecular pathways (Fig. 5d).

## Discussion

In the present analysis, we showed that the molecular landscape of LTS and STS of diffuse gliomas previously classified as WHO grade II and III varies greatly as reflected by integrated classification frameworks and DNA methylation profiling. While we could confirm that most LTS consisted of patients with *IDH-*mutant glioma and most STS had *IDH-*wildtype gliomas and thereby validate the current classification framework, there were outliers with unexpected prognosis based on their molecular background and DNA methylation profiles.

Indeed, ~ 5% of patients with *IDH-*mutant tumors in our cohort had an unexpectedly short OS below 12 months. Furthermore, unsupervised clustering of DNA methylation profiles revealed STS within the cluster primarily consisting of *IDH-*mutant LTS. While some tumors showed copy number alterations including homozygous losses of *CDKN2A/B,* further factors remain largely unknown. In addition to the adverse prognostic impact of homozygous deletions of *CDKN2A/B* in astrocytomas as acknowledged in the current WHO 2021 classification [2], also an association of hemizygous deletions has been described recently [25]. Moreover, mismatch repair deficient *IDH-*mutant astrocytomas are characterized by worse outcomes [4], and *CDKN2A/B* deletions may occur together with other copy number alterations in oligodendroglioma, defining the distinct subgroup of oligosarcomas linked to short survival [5]. Further research in clinical and molecular prognostic factors in *IDH-*mutant gliomas are of high interest given the approval of the IDH inhibitor vorasidenib, opening new therapeutic avenues to postpone tumor recurrence and thereby adverse effects of immediate radiotherapy and/or chemotherapy [26].

Conversely, DNA methylation profiles revealed LTS and MTS within the cluster primarily consisting of STS. Given that the molecular drivers of divergent prognosis remain unclear in some “blackbox” cases, refined prognostic stratification is urgently needed to provide a rational basis for the clinical decision between postoperative treatment modalities [20].

Considerable heterogeneity was observed among *IDH-*wildtype tumors, of whom the majority consisted of STS and was reclassified as glioblastoma based on the presence of molecular markers such as gain of chromosome 7p and loss of 10q, amplification of epidermal growth factor receptor (*EGFR*) and/or *TERT* promoter mutations [2]. Indeed, sampling error in biopsies and the pathological workup might lead to histological undergrading [27], and tumors harboring these molecular alterations were shown to exhibit significantly worse survival than other tumors classified as low-grade gliomas [8, 12, 28]. The cases in our cohort underscore the limited prognosis of “molecular” glioblastoma, as none of the included tumors showed histological grade IV/4 criteria at original diagnosis. On the other hand, some retrospective studies suggest longer PFS and tendentially longer OS in molecular glioblastoma compared to tumors fulfilling histological criteria of glioblastoma as molecular alterations might precede histological signs of necrosis or microvascular proliferation [29, 30]. However, well-annotated large-scale validation is needed to provide a rational basis for clinical trial design and clinical management in these tumors.

Beside glioblastomas, *IDH-*wildtype tumor entities in our cohort included glial and glioneuronal tumors with vastly differing biological behavior. Whereas some tumor types have been incorporated in the 2021 WHO classification, other methylation classes are provisional (such as high-grade diffuse glioma of the midline/posterior fossa: H3/IDH-wildtype; adult-type diffuse high grade neuroepithelial tumor, IDH-wildtype, subtypes B/D) [2]. In these, clinical annotation and molecular phenotyping is scarce, especially in pediatric-type tumors only rarely affecting adults although their occurrence in non-pediatric populations might be underestimated [31]. Besides, small case series of myxoid glioneuronal tumors showed occurrence at all ages and overall benign clinical courses, and *PDGFRA* mutation has been postulated as oncogenic driver [32–34]. The methylation class high-grade diffuse glioma of the midline/posterior fossa, H3/IDH-wildtype involves tumors classified as glioblastomas, but not occurring in the cerebral hemispheres and characterized by distinct methylation profiles, although the further molecular and clinical significance remains unclear [35]. Similarly, novel subtypes of adult-type diffuse high-grade gliomas have distinct characteristics and may be characterized by better prognosis compared to glioblastomas according to retrospective case series [36].

Functional enrichment analyses of novel subtypes revealed differentially methylated CpG sites of genes involved in Hippo and synaptic/neurotransmitter signaling pathways. Recently, enriched gene sets of Hippo signaling were also described in LTS of glioblastomas based on their DNA methylation profile [37], and expression of Hippo pathway members has been described to correlate with tumor grade in astrocytoma and invasiveness of glioblastoma [38, 39]. Moreover, it has been shown that glutamatergic signaling induces calcium currents eventually stimulating glioma growth in preclinical models, and inhibition of postsynaptic AMPA receptors by antiepileptic drugs such as perampanel might decrease tumor proliferation [40–43]. While our results support the hypothesis that these pathways may also be involved in rare CNS tumors, further investigation in vitro and in vivo is needed to validate these findings. Indeed, drug repurposing trials investigating the inhibition of glutamatergic signaling in glioblastoma are ongoing and will shed light on the actionability of “neuro-tumoral” synapses as treatment targets [44, 45].

Certainly, our study has inherent limitations. The retrospective cohort of diffuse gliomas diagnosed in a period of 20 years is associated with heterogeneous treatment patterns over time and between participating centers as well as missing clinical information. Moreover, the extent of resection has partly been retrieved from surgical reports in patients where postoperative imaging was not available, limiting the reliability of this information [46]. Due to limited documentation, all-cause death was used as survival endpoint, challenging the validity of cancer-associated mortality in our cohort. In addition, DNA methylation analyses and full molecular reclassification according to the WHO 2021 classification including *CDKN2A/B* status were only feasible in a subset of patients given the limited availability of FFPE tissue, and low cellularity in tumor samples with resulting low yields of tumor DNA may compromise the validity of methylation profiles.

## Conclusions

Our study underscores the utility of refined CNS tumor classification frameworks and DNA methylation profiling as they result in clinically more homogenous tumor entities in a real-life setting. Further integration of novel, molecularly defined subgroups with clinical annotation and prognostic information is needed, ideally within prospective, multicentric registries. Besides supporting clinical decision-making and counseling of patients, integrated information allows to facilitate the development of novel treatment strategies and optimize clinical trial design in rare entities of CNS tumors.

## Supplementary Information

Below is the link to the electronic supplementary material.

Supplementary file1 (PDF 618 KB)

Supplementary file2 (PDF 115 KB)

## Data Availability

Underlying data can be provided upon reasonable request to the corresponding author and approval from relevant regulatory authorities.
