# Supplementary material for: Clinical characteristics, molecular reclassification trajectories and DNA methylation patterns of long- and short-term survivors of WHO grade II and III glioma
Source: J Neurol. 2025 Feb 15;272(3):210. doi: 10.1007/s00415-025-12923-6 (PMC11829921; doi:10.1007/s00415-025-12923-6)
Supplement: Supplementary file 1 — Supplementary file1 (PDF 618 KB) [file 415_2025_12923_MOESM1_ESM.pdf]

Supplementary Figures

Supplementary Fig. 1. Relative frequency of symptoms in long- and short-term survivors. (A) Sensoric deficits, (B) visual disturbances, (C) behavioral changes, (D) ataxia, (E) vertigo, (F) new-onset headache, (G) elevated intracranial pressure.

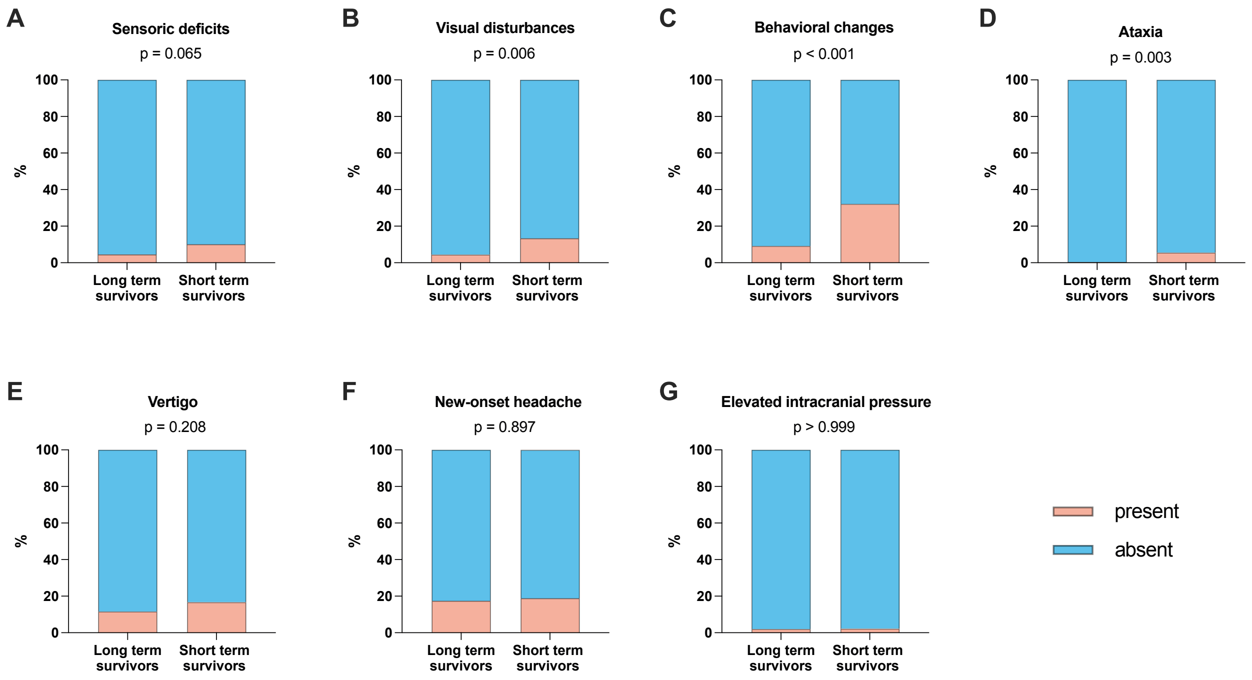

Supplementary Fig. 2. Molecular reclassification of tumors according to (A) WHO 2016 and WHO 2021 classifications. Other including anaplastic pilocytic astrocytoma, dysembryoplastic neuroepithelial tumor and diffuse midline glioma, *H3K27*-altered. LTS = long-term survivors, NOS = not otherwise specified, STS = short-term survivors

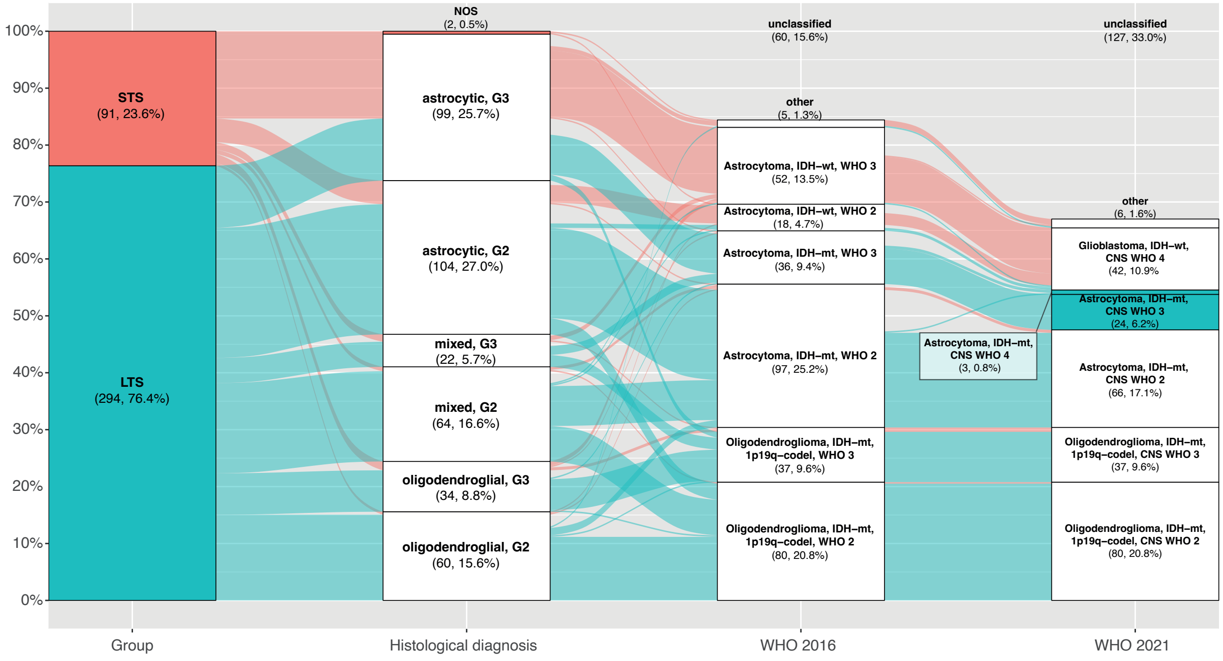

Supplementary Fig. 3. Survival analysis in *IDH*-mutant astrocytomas of the DNA methylation profiling cohort according to CNV load. Cutpoint = 643 MBp (mega base pairs) according to optimal cutpoint calculation using surv_cutpoint. P value as determined by log-rank test.

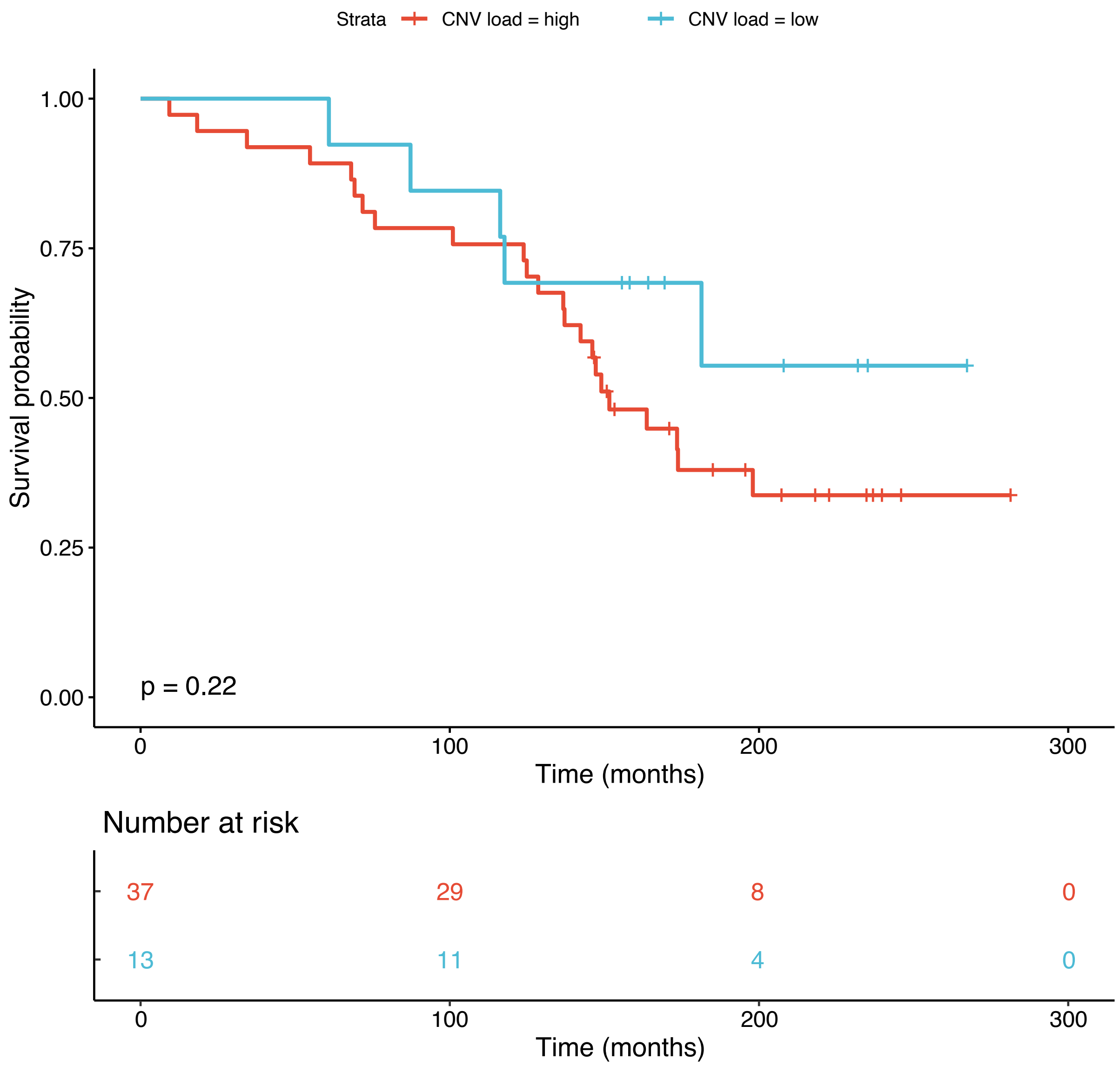

Supplementary Fig. 4. Heatmap and clustering analysis based on DNA methylation profiling of rare subgroups including (A) diffuse high-grade neuroepithelial tumor (adult-type non defined types B/D); (B) diffuse pediatric-type high-grade glioma; H3 wildtype and IDH wildtype subtype A & B (novel); (C) high-grade diffuse glioma of the midline/posterior fossa; H3/IDH-wildtype; (D) myxoid glioneuronal tumor, PDGFRA-mutant. Control groups are (A) *IDH*-mutant gliomas as reference group for long-term survivors and (B/C/D) glioblastoma (*IDH*-wildtype) as reference group for short-term survivors.

(follows on next page)

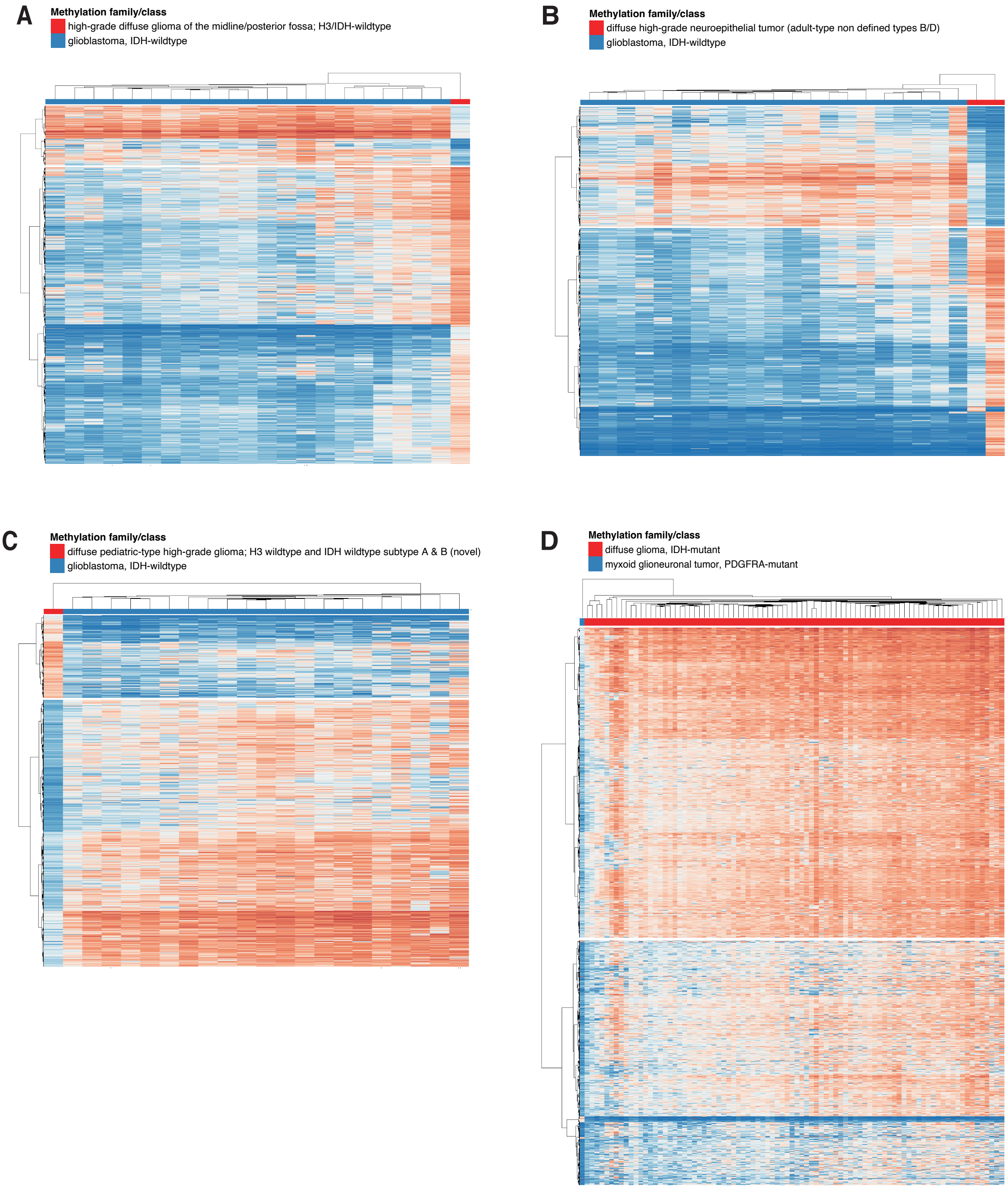
A
Methylation family/class
high-grade diffuse glioma of the midline/posterior fossa; H3/IDH-wildtype
glioblastoma, IDH-wildtype
B
Methylation family/class
diffuse high-grade neuroepithelial tumor (adult-type non defined types B/D)
glioblastoma, IDH-wildtype
C
Methylation family/class
diffuse pediatric-type high-grade glioma; H3 wildtype and IDH wildtype subtype A & B (novel)
glioblastoma, IDH-wildtype
D
Methylation family/class
diffuse glioma, IDH-mutant
myxoid glioneuronal tumor, PDGFRA-mutant
