# Supplementary material for: Clinical characteristics, molecular reclassification trajectories and DNA methylation patterns of long- and short-term survivors of WHO grade II and III glioma
Source: J Neurol. 2025 Feb 15;272(3):210. doi: 10.1007/s00415-025-12923-6 (PMC11829921; doi:10.1007/s00415-025-12923-6)
Supplement: Supplementary file 2 — Supplementary file2 (PDF 115 KB) [file 415_2025_12923_MOESM2_ESM.pdf]

Supplementary Tables

Supplementary Table 1. Baseline characteristics of DNA methylation profiling cohort.

	**n = 147**
**Group**	
- long term survivors (LTS)	83 (56.5%)
- short term survivors (STS)	43 (29.3%)
- medium term survivors (MTS)	21 (14.3%)
**Sex**	
- female	79 (53.7%)
- male	68 (46.3%)
**Median age (years, range) at diagnosis**	43 (20 – 81)
**Median Karnofsky Performance Status (range)**	90% (40%-100%)
**Original histological diagnosis at first surgery (including pre-WHO 2016 diagnoses)**	
- astrocytic	77 (52.4%)
- oligodendroglial	39 (26.5%)
- mixed	30 (20.4%)
- not otherwise specified (NOS)	1 (0.7%)
**WHO grade at first surgery**	
- WHO grade II	73 (49.7%)
- WHO grade III	74 (50.3%)
**Tumor site**	
- Frontal	67 (45.6%)
- Temporal	45 (30.6%)
- Parietal	12 (8.2%)
- Occipital	3 (2.0%)
- Insular	8 (5.4%)
- Other (supratentorial)	8 (5.4%)
- Other (infratentorial)	4 (2.7%)
**Extent of resection at first surgery**	
- Gross total resection (GTR)	53 (36.1%)
- Subtotal resection (STR)	60 (40.8%)
- Biopsy	31 (21.1%)
- *unknown*	*3 (2.0%)*
**Postoperative treatment**	
- Radio-chemotherapy	41 (27.9%)
- Radiotherapy only	26 (17.7%)
- Chemotherapy only	10 (6.8%)
- Watch-and-wait	70 (47.6%)

Supplementary Table 2. Patients' characteristics in distinct methylation classes.

Group	Methylation class	n	Age at diagnosis (median, range)	Sex		Tumor location	Original histological diagnosis	Extent of resection	Adjuvant treatment	Notes
				M	F					
LTS	Diffuse glioma, *IDH*-mutant and 1p19q co-deleted [oligodendroglial type]	41	39 (23 – 59)	18 (43.9%)	23 (56.1%)	Frontal: 26 (63.4%)<br>Temporal: 9 (22.0%)<br>Parietal: 2 (4.9%)<br>Insular: 2 (4.9%)<br>Occipital:1 (2.4%)<br>Other (supratent.): 1 (2.4%)	Astrocytic, G2: 5 (12.2%)<br>Astrocytic, G3: 2 (4.9%)<br>Oligodendroglial, G2: 12 (29.3%)<br>Oligodendroglial, G3: 12 (29.3%)<br>Mixed, G2: 7 (17.1%)<br>Mixed, G3: 3 (7.3%)	GTR: 19 (46.3%)<br>STR: 16 (29.0%)<br>Biopsy: 5 (12.2%)<br>Unknown: 1 (2.4%)	R-ChT: 11 (26.8%)<br>RT: 8 (19.5%)<br>ChT: 1 (2.4%)<br>None: 21 (51.2%)	
	Diffuse glioma, *IDH*-mutant and 1p19q retained [astroglial type], low grade	34	34.5 (21 – 52)	15 (44.1%)	19 (55.9%)	Frontal: 16 (47.1%)<br>Temporal: 10 (29.4%)<br>Parietal: 3 (8.8%)<br>Insular: 3 (8.8%)<br>Occipital: 2 (5.9%)	Astrocytic, G2: 14 (41.2%)<br>Astrocytic, G3: 9 (26.5%)<br>Oligodendroglial, G2: 2 (5.9%)<br>Mixed, G2: 7 (20.6%)<br>Mixed, G3: 2 (5.9%)	GTR: 17 (50.0%)<br>STR: 14 (41.2%)<br>Biopsy: 3 (8.8%)	R-ChT: 5 (14.7%)<br>RT: 7 (20.6%)<br>ChT: 2 (5.9%)<br>None: 20 (58.8%)	
	Diffuse glioma, *IDH*-mutant and 1p19q retained [astroglial type], high grade	3	27 (25 – 36)	1 (33.3%)	2 (66.7%)	Frontal: 2 (66.7%)<br>Insular: 1 (33.3%)	Astrocytic, G3: 3 (100.0%)	GTR: 2 (66.7%)<br>Biopsy: 1 (33.3%)	R-ChT: 1 (33.3%)<br>RT: 1 (33.3%)<br>ChT: 1 (33.3%)	
	Myxoid glioneuronal	1	27	1 (100.0%)	-	Temporal	Oligodendroglial, G2	GTR	None	Dysembryoplastic neuroectodermal tumor

	tumor, *PDGFRA*-mutant								(DNET) according to MC 11b6
MTS	Diffuse glioma, *IDH*-mutant and 1p19q retained [astroglial type], low grade	11	33<br>(20 – 74)	5<br>(45.5%)	6<br>(54.5%)	Temporal: 6 (54.5%)<br>Frontal: 4 (36.4%)<br>Parietal: 1 (9.1%)	Astrocytic, G2: 5 (45.5%)<br>Astrocytic, G3: 1 (9.1%)<br>Mixed, G2: 3 (27.3%)<br>Mixed, G3: 2 (18.2%)	GTR: 5 (45.5%)<br>STR: 5 (45.5%)<br>Biopsy: 1 (9.1%)	R-ChT: 2 (18.2%)<br>RT: 2 (18.2%)<br>None: 7 (63.6%)	
	Diffuse glioma, *IDH*-mutant and 1p19q co-deleted [oligodendroglial type]	6	42.5<br>(31 – 70)	3<br>(50.0%)	53<br>(50.0%)	Frontal: 4 (66.7%)<br>Temporal: 2 (33.3%)	Astrocytic, G2: 1 (16.7%)<br>Oligodendroglial, G3: 4 (66.7%)<br>Mixed, G3: 1 (16.7%)	GTR: 4 (66.7%)<br>STR: 1 (16.7%)<br>Biopsy: 1 (16.7%)	R-ChT: 4 (66.7%)<br>ChT: 1 (16.7%)<br>None: 1 (16.7%)	
	Diffuse glioma, *IDH*-mutant and 1p19q retained [astroglial type], high grade	1	35	1<br>(100.0%)		Temporal	Astrocytic, G3	STR	R-ChT	
STS	Glioblastoma, *IDH*-wildtype, mesenchymal type	9	69<br>(54 – 81)	5<br>(55.6%)	4<br>(44.4%)	Temporal: 4 (44.4%)<br>Frontal: 2 (22.2%)<br>Parietal: 2 (22.2%)<br>Insular: 1 (11.1%)	Astrocytic, G2: 1 (11.1%)<br>Astrocytic, G3: 8 (88.9%)	GTR: 1 (11.1%)<br>STR: 5 (55.6%)<br>Biopsy: 3 (33.3%)	R-ChT: 6 (66.7%)<br>RT: 1 (11.1%)<br>None: 2 (22.2%)	
	Glioblastoma, *IDH*-wildtype, RTK2 type	8	71.5<br>(48 – 78)	4<br>(50.0%)	4<br>(50.0%)	Frontal: 3 (37.5%)<br>Parietal: 2 (25.0%)<br>Temporal: 1 (12.5%)<br>Other (infrat.): 1 (12.5%)<br>Other (supratent.): 1 (12.5%)	Astrocytic: G2: 1 (12.5%)<br>Astrocytic, G3: 6 (75.0%)<br>Mixed, G3: 1 (12.5%)	GTR: 1 (12.5%)<br>STR: 3 (37.5%)<br>Biopsy: 4 (50.0%)	R-ChT: 2 (25.0%)<br>RT: 1 (12.5%)<br>ChT: 1 (12.5%)<br>None: 4 (50.0%)	

	Glioblastoma, *IDH*-wildtype, RTK1 type	4	60 (43 – 72)	3 (75.0%)	1 (25.0%)	Temporal: 3 (75.0%) Frontal: 1 (25.0%)	Astrocytic, G2: 1 (25.0%) Astrocytic, G3: 3 (75.0%)	GTR: 1 (25.0%) STR: 1 (25.0%) Biopsy: 2 (50.0%)	R-ChT: 2 (50.0%) None: 2 (50.0%)	
	Diffuse glioma, *IDH*-mutant and 1p19q co-deleted [oligodendroglial type]	3	49 (30 – 59)	-	3 (100.0%)	Frontal: 2 (66.7%) Temporal: 1 (33.3%)	Oligodendroglial, G2: 1 (33.3%) Oligodendroglial: G3: 1 (33.3%) Mixed, G3: 1 (33.3%)	GTR: 1 (33.3%) STR: 2 (66.7%)	ChT: 1 (33.3%) None: 2 (66.7%)	
	High-grade astrocytoma with piloid features	2	56 (36 – 76)	2 (100.0%)	-	Other (infrat.): 1 (50.0%) Temporal: 1 (50.0%)	Astrocytic, G3: 2 (100.0%)	Biopsy: 2 (100.0%)	R-ChT: 1 (50.0%) RT: 1 (50.0%)	
	Diffuse glioma, *IDH*-mutant and 1p19q retained [astroglial type], high grade	1	66	1 (100.0%)	-	Frontal	Oligodendroglial, G3	GTR	R-ChT	IDH glioma, subclass 1p/19q codeleted oligodendroglioma according to MC 11b6; CNV profile showing 1p/19q codeletion as well as partial deletion of chromosome 9 (including loss of *CDKN2A/B).*
	Medulloblastoma, SHH-activated, subtype 2	1	27	-	1 (100.0%)	Other (suprat.)	Astrocytic, G3	Biopsy	R-ChT	Diffuse midline glioma, H3K27m-altered according to MC 11b6; IHC for H3K27M mutation positive
	High-grade diffuse glioma of the midline/posterior fossa: H3/*IDH*-wildtype	1	76	1 (100.0%)	-	Other (infrat.)	Astrocytic, G3	STR	None	
	Diffuse high-grade neuroepithelial tumor (adult-type, non-defined type B)	1	64	1 (100.0%)	-	Frontal	Oligodendroglial, G3	STR	RT	
	Diffuse high-grade neuroepithelial tumor (adult-type,	1	59	1 (100.0%)	-	Temporal	NOS	STR	None	

	non-defined type D)									
	Diffuse pediatric-type high-grade glioma, H3 wildtype and *IDH* wildtype, subtype A&B (novel)	1	69	-	1 (100.0%)	Other (suprat.)	Mixed, G3	STR	RT	
	Inflammatory microenvironment	1	81	-	1 (100.0%)	Parietal	Astrocytic, G3	Biopsy	None	
